# Engineering of Exosomes to Target Cancer Metastasis

**DOI:** 10.1007/s12195-019-00607-x

**Published:** 2019-12-23

**Authors:** Zhenjiang Zhang, Jenna A. Dombroski, Michael R. King

**Affiliations:** Department of Biomedical Engineering, Vanderbilt University, Nashville, TN 37212 USA

## Abstract

As a nanoscale subset of extracellular vehicles, exosomes represent a new pathway of intercellular communication by delivering cargos such as proteins and nucleic acids to recipient cells. Importantly, it has been well documented that exosome-mediated delivery of such cargo is involved in many pathological processes such as tumor progression, cancer metastasis, and development of drug resistance. Innately biocompatible and possessing ideal structural properties, exosomes offer distinct advantages for drug delivery over artificial nanoscale drug carriers. In this review, we summarize recent progress in methods for engineering exosomes including isolation techniques and exogenous cargo encapsulation, with a focus on applications of engineered exosomes to target cancer metastasis.

## Introduction

Extracellular vesicles (EVs) were first described by Trams *et al.* in 1981 as cell-secreted particles that carried membrane-bound enzymes, and could be taken up by recipient cells.^112^ The authors keenly predicted that EVs could represent an important pathway to transfer information between cells and might be developed to package and deliver therapeutic molecules like structurally similar liposomes. However, initially EVs were more widely regarded as “garbage bags” for disposal of undesired cellular components.^116^ A subset of extracellular vesicles in the 30–150 nm range, which are released from cells upon fusion of an intermediate endocytic compartment called the multivesicular body (MVB) with the plasma membrane, were later defined as exosomes.^93^ Exosomes were subsequently found to be specialized for intercellular signaling by carrying proteins, nucleic acids, lipids and metabolic cargo from source cells to neighboring recipient cells or even to distant organs.^75^

Exosomes facilitate effective intercellular communication that can regulate cellular functions such as proliferation, apoptosis and migration.^40^ Mounting studies support the understanding of exosomes as key players in tumor growth.^40^,^72^ In fact, cancer cells have been found to secrete more exosomes than noncancerous cells.^6^ Over the last decade, exosomes shed by cancer cells have been found to facilitate metastasis, which accounts for over 90% of cancer-related deaths.^101^,^123^,^126^,^127^,^141^ Metastasis occurs when a cancer cell derived from a primary tumor intravasates into the bloodstream in the form of a circulating tumor cell, which has the potential to grow into a secondary tumor following extravasation.^114^ Evidence has supported that exosomes play a critical role in several steps in the metastatic process.^141^ As a result, exosomes have become an increasingly important research target for the prevention of metastasis.^127^ Anti-metastatic treatments that have attracted intensive research efforts include immunotherapy such as chimeric antigen receptor T (CAR T) cells or TRAIL-coated leukocytes as well as stem cell and virotherapy.^78^,^87^,^113^,^121^

Exosomes have been pursued as a delivery vehicle for a variety of therapeutics for targeted treatment.^7^,^11^,^69^,^73^,^104^,^132^ Compared to artificial nanoscale vehicles, exosomes possess a number of advantages that can be exploited. For one, exosomes naturally deliver their membrane and cytoplasm components by fusing with the target cell membrane.^7^ Exogenous therapeutics can thus be encapsulated in exosomes and delivered in a hitchhiking manner. In addition, exosomes, particularly those collected from patient tissues or blood, possess low immunogenicity and thus intrinsic long-term circulatory capability, and excellent biocompatibility.^64^ Several studies also suggest that exosomes secreted by specific cell types exhibit a very specific cell tropism, supporting highly targeted cargo delivery.^25^,^54^

Our growing understanding of the biology of exosomes and experience in engineering exosomes for diagnostic or therapeutic purposes have provided promising potential for the treatment of tumor metastases.^13^,^22^,^68^ In this review, we discuss the recent advances concerning the engineering of exosomes to target metastasis, with a focus on the methods of exosome isolation and engineering, and therapeutic effects of engineered exosomes for antimetastatic therapy. We will only briefly introduce the biogenesis, structure, and contents of exosomes, and their roles in cancer, as several existing review articles have covered these topics.^7^,^11^,^40^,^64^,^73^,^75^,^93^,^104^,^116^,^132^

### Biogenesis

**Figure 1:**
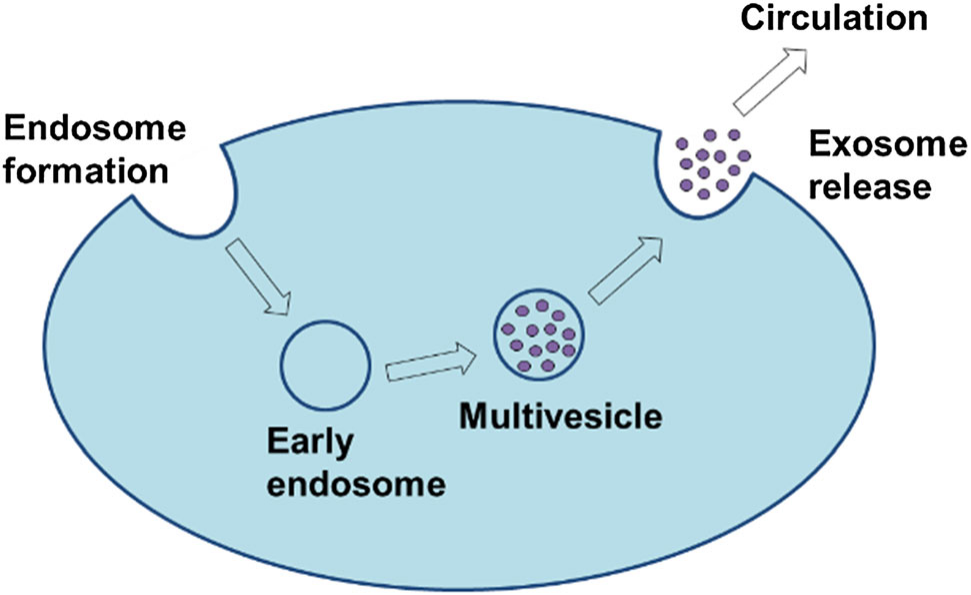
Exosome biogenesis. Formation of exosomes begins with membrane invagination in the form of an endosome, leading to the development of the early endosome. Upon maturation, the endosome becomes a multivesicular endosome, which releases its contents in the form of exosomes.

Exosomes are defined as extracellular vesicles originating from the exocytosis of multivesicular endosomes (MVEs) from the plasma membrane of a cell.^35^ This exosome biogenesis was discovered by two groups of researchers in the 1980s, with papers published within a week of each other.^34^,^86^ The process, applied to maturing reticulocytes at the time, was eventually found to be applicable across all cell types.^34^ During this biogenesis process, the plasma membrane invaginates to form an early endosome. Upon maturation into MVE containing proteins, the endosome will either be degraded by the lysosome or fused back into the plasma membrane.^86^ Its exocytosis from the membrane results in the release of the exosome into circulation^115^ (Fig. 1).

### Structure and Contents

While exosomes possess similar morphological qualities to other EVs in their spherical or spheroidal shapeandan enclosed lipid bilayer membrane, they have unique features such as size, density, and composition.^108^ Exosomes can be distinguished from other EVs by size, with a diameter ranging from 30 to 150 nm.^108^ In addition, the density of exosomes is between 1.15 and 1.19 g/mL, which allow them to float on a continuous sucrose gradient.^109^

The composition of exosomes includes proteins, nucleic acids, lipids and metabolic cargo.^38^ Proteins found in exosomes are limited in range, derived primarily from the cytosol.^38^ Proteins include those related to the endocytic pathway, as well as adhesion and targeting proteins. Many of these are membrane bound proteins, originating from the invagination of the membrane that produces the exosomes.

Given that exosomes stem from the invagination of the plasma membrane, exosomes are composed of lipids consistent with the lipid bilayer of their parental cells. Consequently, they possess a similar fraction of the membrane phospholipid PE (phosphatidylethanolamine) in exosomes as their parent cells.^97^

### Characterization Techniques

The characterization of exosomes can be performed visually, by using various dynamic light scattering (DLS) and microscopy techniques, and flow cytometry. Due to the small size range of exosomes, scanning electron microscopy (SEM) and transmission electron microscopy (TEM) are frequently used to visualize exosomes *via* negative staining. Size distribution and concentration, as well as sample purity, can be measured with DLS using a Zetasizer instrument (Malvern) and nanoparticle tracking analysis (NTA, Malvern).^130^ Labelling exosomes with lipophilic membrane dyes such as PKH-26 or PKH-67 enables analysis *via* flow cytometry.^59^ Fluorescence visualization is aided by the metabolic labeling of exosomes, using fatty acid analogues.^21^ In conjunction with such labeling, Western blot can be used to analyze specific proteins such as heat shock protein 90 (HSP90), ALG-2-interacting protein X (Alix), Tumor susceptibility gene 101 (TSG101), and the EV related tetraspanin protein (CD63).^21^

## Role of Exosomes in Metastasis

It has been shown that cancer cells secrete more exosomes than their non-cancerous counterparts.^111^ Cancer-derived exosomes have been reported to promote metastasis in a variety of ways including altering the immune system, promoting epithelial to mesenchymal transition (EMT), organotropism, and angiogenesis. These phenomena were observed particularly in exosomes derived from metastatic cancer cells, which were found to transfer their host cell’s invasive properties to non-metastatic cancer cells.^36^

### Altering the Immune System

Exosomes have been found to exhibit the ability to induce immune suppression.^139^ For instance, tumor-derived exosomes upregulate specific immunosuppressive factors such as GM-CSF and TNF-α.^43^ They also impair the ability of natural killer (NK) cells to carry out cytotoxic functions by secreting TGF-1β or blocking IL-2 mediated activation.^8^ Exosomes can contain FAS and TRAIL ligands to induce the death-receptor activated killing of lymphocytes, extending their immune-modulating effects.^4^ The effect of tumor exosomes on cellular immunity has also been studied. Tumor exosomes have been found to reduce cytotoxic T cell (CD8^+^) counts.^125^ Additionally, exosomes can promote the conversion of helper T cells (CD4^+^) into regulatory T cells.^124^ The upregulation of T_regs_ aids in the ability of the tumor microenvironment to suppress and evade an immune system response. T cell activation has also been inhibited by exosomes.^107^ The mechanism in which they are able to achieve this inhibition is *via* targeting TGF-β.^92^ Similarly, exosomes released from metastatic melanomas have been found to carry surface programmed death-ligand 1 (PD-L1), which aids in tumor growth by inhibiting CD8^+^ T cell function.^15^

### Promoting EMT

The process of EMT occurs when tissue epithelial cells possess altered biochemical factors that leave them with a more mesenchymal phenotype, aiding in functions such as migration and invasion.^49^ Exosomes released from cancer cells have been found to directly promote the EMT process by their delivery contents. For instance, cancer cell-derived exosomes can contain high amounts of TGF-β, caveolin-1, HIF-1α, β-catenin, LMP1 and H1H1α, which results in a more invasive phenotype for receiving cells.^41^ miR-21 and matrix metalloproteinase-13 can also be enriched in these exosomes, which enhance mesenchymal markers such as vimentin and suppresses epithelial markers like E-cadherin.^94^ EMT can also be triggered indirectly when cancer-associated fibroblasts (CAFs) release exosomes that convert mesenchymal stem cells to fibrous-associated fibroblasts in the pericellular microenvironment.^122^

### Metastatic Organotropism

Organotropism is defined as the non-random process which results in distant metastasis to specific organs.^68^ Exosomes derived from tumors present integrins that helps drive organotropic properties.^44^ This had been observed from breast cancer exosomes moving to lung tissue in an orthotopic mouse model, as well as in pancreatic ductal adenocarcinoma.^22^ Specifically, MDA-MB-231 breast cancer cell-derived exosomes were shown to exhibit metastatic homing to the lungs and brain regardless of whether they were injected *via* tail vein, intracardial or retro-orbital route.^44^

### Angiogenesis

Angiogenesis and vascular permeability are activated and upregulated by exosomes derived from cancer cells. These exosomes carry pro-angiogenic factors such as VEGF, TIMP-1, IL-6, and FGF and cause their upregulation in recipient cells.^106^ In addition, they carry paracrine signaling factors and mRNAs to alter the genetics and drive genetic expression toward angiogenesis.^96^ Angiogenesis- and metastasis-promoting microRNAs such as miR-9, miR-23a, and miR-210 have been found as exosome cargo.^13^ Tumor-derived exosomes, as well as exosomes derived from cancer associated fibroblasts (CAFs), release factors that recruit and activate endothelial progenitor cells.^96^

## Exosome Isolation Techniques

One crucial step in the study of exosomes is to isolate exosomes from a complex mixture of cell culture media, tissues or bodily fluids that contains cells, cell debris, other particulate components and macromolecules. An optimal method for exosome isolation is expected to exhibit high recovery yield and high purity of exosomes, and high efficiency as well. Several isolation techniques have been utilized in published exosome studies, each exploiting particular properties of exosomes, such as their density, shape, size, and unique surface proteins to aid their isolation (Table 1).

**Table 1:** Summary of exosome isolation techniques.

Isolation techniques	Isolation principles	Advantages	Disadvantages	References
Centrifugation	Density, size and shape	High yield, low cost and scalability	Time-consuming, subject to equipment variability	26,57,61,65,129
Size-based Techniques	Size	Fast, high purity, moderate scalability	Moderate yield, subject to clogging and loss due to column or filter attachment	57,62,66
Immunoaffinity	Specific interaction between antibody and antigen	Fast, high purity	Low yield, high possibility of subtyping	82,134,140
Polymer precipitation	Solubility and dispersity	Easy to use, high scalability	Subject to protein contamination	10,37,48,90
Microfluidic separation	Various properties incorporated into microfluidic channels	Fast, easy integration with other techniques	Limited to small sample volume, requires in-house made microfluidic devices	16,19,32,45,83,99

### Differential Ultracentrifugation and Density Gradient Centrifugation

Ultracentrifugation is considered the “gold standard” for exosome isolation and makes up > 50% of all exosome isolation techniques utilized in reported exosome research.^129^ Sequential centrifugation is performed in a typical isolation experiment to remove other components until only the exosomes remain. First, a low speed centrifugation step (~ 400×*g*) is performed to remove cells and large cellular debris from an exosome containing sample, e.g., conditioned cell culture media or biological fluid. Next to be removed are the smaller debris and intact organelles at 10,000–20,000×*g*. The final step is ultracentrifugation (100,000–150,000×*g*) of the supernatant to form a pellet of exosomes.^65^ Due to the heterogeneity of exosomes and considerable overlap in size with other EVs, exosomes isolated by differential ultracentrifugation are often found to be contaminated by other EVs, protein aggregates or even large individual proteins.^26^ One resolution to this challenge is to remove the contaminants from the isolated exosomes with gradient density centrifugation which uses a continuous sucrose gradient to separate exosomes from contaminants based on their difference in densities.^57^ This technique has been known to improve the purity of exosome isolates. However, gradient centrifugation is notorious for being time consuming, ~ 80 h in the case of exosome purification.^61^ Also, this method is subject to equipment-dependent variability because minimal differences of centrifugation factors such as rotor type, angle and radius can result in variations in the type, purity and yield of exosomes isolated.^23^

### Size-Based Filtration, Chromatography and Fractionation

Ultrafiltration, including syringe-driven filtration, may be the most straightforward method for exosome isolation.^31^ Exosomes can be separated from other components in the sample using membrane filters with defined pore size or molecular weight (MW) limits. Due to the size heterogeneity of the components in exosome-containing samples, sequential filtration is needed to remove other components that are significantly larger or smaller than exosomes.^62^ The most common pore sizes for filtration membranes are 0.8, 0.45 or 0.22 µm.^130^

A commonly used protein concentrator has been reported to concentrate exosomes from urine samples with high yield and efficiency.^77^ The samples were first centrifuged at 17,000×*g* to remove particles much larger than exosomes before being concentrated with a concentrator with a uniform pore size of 13 nm or a ~ 100 kDa MW cutoff (MWCO).^77^

For exosome isolation from cell culture supernatants, a 3-step sequential filtration has been reported. The first step is to remove floating cells and large cell debris using a 100-nm polyethersulfone (PES) filter.^61^ To remove components smaller than exosomes, the filtrate is then subjected to tangential flow filtration with 500 kDa MWCO hollow fiber PES filters. The retentate collected is then dialyzed to further remove contaminants as completely as possible before finally being filtered with a 100 nm polycarbonate track-etched filter.^61^

Exosome isolation by ultrafiltration is much faster than that by ultracentrifugation and can be completed without using any other special equipment. However, exosomes may partly become deformed or broken when they are forced through nanoscale filters.^61^

Size exclusion chromatography (SEC) is a promising method for exosome isolation because of its capability to separate nanoscale particles based on their hydrodynamic size.^57^ The SEC column is packed with porous beads so that components with a smaller size have to go through many small pores before being eluted out of the column while larger components can pass the beads faster by avoiding entering the pores.^39^ Exosomes in mesenchymal stem cell (MSC)-conditioned medium were reported to be successfully isolated by SEC.^81^ As examined by TEM, the isolated exosomes are structurally intact.^81^ The major advantage of using SEC for exosome isolation is that the technique preserves the structural integrity and biological activity of exosomes while other components are removed. Moreover, SEC is a very sensitive method for exosome isolation and exhibits high reproducibility.^7^,^61^ While SEC is commonly driven by gravity flow, which is a time-consuming process, it can be sped up by incorporation of a SEC column with HPLC or Fast Protein Liquid Chromatography (FPLC).^39^ In addition, SEC can be coupled with ultracentrifugation to concentrate the final exosome dispersion.^66^

Another size-based separation technique that has been applied to exosome isolation is Asymmetric Flow Field-Flow Fractionation (AF4).^137^ AF4 is a fractionation method that is commonly used for the separation/analysis of polymers, proteins and nanoparticles.^30^ Fractionation in AF4 takes place in a thin chamber, in which a laminar flow carries a sample through the chamber and a crossflow separation field pushes the particulate components towards the accumulation wall of the chamber. Brownian motion of the flowing particles in the sample thus interferes with the crossflow against the accumulation wall in a diffusivity-dependent manner. Smaller particles that diffuse faster are reflected back into the faster flowing center from the accumulation wall and are eluted earlier than larger ones. Successful isolation using AF4 has been reported to isolate exosomes from B16-F10 mouse melanoma cell culture into vesicle subpopulations by size.^51^ In another AF4 study of exosomes, two major factors on fractionation quality of exosomes were identified to be cross-flow conditions and the channel thickness, while the focusing time showed less significant impact. Also, the exosomes were found to be eluted together with a population of smaller vesicle-like particles, as revealed by online UV and multi-angle light scattering (MALS), and subsequent DLS analysis.^95^ While possessing the potential to greatly facilitate exosome research and application, AF4 does require specialized facilities and operation expertise.

### Immunoaffinity

Surface proteins and other molecules that are unique to, or highly concentrated on, exosomes in exosome-containing samples offer opportunities for specific isolation by designing antibody-mediated immunoaffinity interaction.^5^ Such surface molecules that have been identified include tetraspanin, TSG101, Alix, annexin, EpCAM and Rab5.^140^ In the immunoaffinity methods, antibodies against the surface markers are immobilized on the surface of beads, filters or other matrices to allow exosomes to bind the matrices specifically. After washing off the unbound fraction, the bound fraction will then be collected by detaching the exosomes from the stationary phase.^65^ Because this technique is based on highly specific antibody recognition, the exosomes obtained are often found to be more pure than those isolated by other methods which are based on their less unique physical properties.^134^ However, there are a few drawbacks of note to this isolation method. First, only a subset of all exosomes express the surface markers and can thus be captured, resulting in a low yield.^7^ In addition, recovering fully intact exosomes can be difficult after antibody binding in immunoaffinity isolation.^82^

Zarovni *et al.* evaluated several commercially available kits for immunoaffinity-based isolation and modified their protocols to increase the purity of exosomes obtained.^136^ Antibodies specific to several distinct exosome surface proteins have been evaluated to identify desirable molecular targets for total exosome capture. Furthermore, the authors incorporated downstream steps allowing on-line quantification and analysis of bound exosomes, which enabled rapid quantification and validation of subpopulations of exosomes with manifold yield.^136^ It has been reported that the optimized assays exhibited high sensitivity which can downscale working plasma volumes to as little as 100 μL.

The efficiency of immunoaffinity-based isolation may be further improved by using antibody-modified magnetic beads.^48^ After incubation with exosome-containing samples, magnetic beads are subjected to a magnetic field to separate the beads out of the sample. Zarovni *et al.* evaluated the feasibility of magnetic beads for isolating exosomes. As little as 1.0 mL of cell culture supernatant can be handled with a similar capture efficiency to that of ultracentrifugation.^48^

Compared to ultracentrifugation for exosome isolation from cell culture medium, immunoaffinity-based isolation exhibits comparable yield with higher purity and advantages of ease of operation and much higher efficiency.^61^ For exosome isolation from a large volume of plasma sample, the yield achieved by magneto-immunocapture was found to be an order of magnitude higher than that obtained by ultracentrifugation.^37^

### Polymer Precipitation

Exosomes can also be isolated *via* a so-called polymer-based precipitation method, which is a widely used method to precipitate viruses and other macromolecules.^10^ Typically, exosome-containing samples are incubated with a precipitation solution containing polyethylene glycol (PEG) with a (MW) of 8000 Da at 4 °C overnight to sequester water molecules and force less soluble components out of solution. The mixture is then subjected to centrifugation at a low speed to pellet the precipitated exosomes.^90^

Compared to other methods of isolation, polymer-based precipitation is easy to use, scalable for large sample volumes and does not require any specialized equipment or lengthy run time.^16^ One weakness of this method is that the exosomes obtained are often found to be contaminated by proteins, subcellular particles and polymer materials.^99^ Additional steps before or after isolation may be used to address this issue. Subcellular particles such as lipoproteins may be removed by centrifugation before isolation, while the polymer may be removed using SEC.^83^

### Microfluidic Separation

As a rapidly-growing engineering field, microfluidics has been widely used for the separation of particles ranging from nanoscale to microscale such as cells and nanoparticles.^19^ It represents a promising solution that can be incorporated with various up-to-date separation and sensing mechanisms for exosome isolation and analysis. Although still at an early-stage of development, microfluidics-based isolation methods hold great promise for translation into the clinic as they typically require a very small volume of samples and yield highly pure exosomes with minimal processing time.^45^ Microfluidics-based technologies for exosome isolation are typically used for diagnostic purposes due to their high sensitivity but limitation in processed sample volume.^32^

Techniques that have been incorporated in microdevices for exosome isolation include immunoaffinity, sieving, and trapping exosomes on porous structures.^19^ Similar to the macroscale immunoaffinity-based method for exosome isolation, antibodies can also be immobilized to the surface of the channels in microfluidic devices for microscale isolation of exosomes.^17^ Multiple groups have described approaches that incorporate microfluidics and immunoaffinity to isolate exosomes and microvesicles, highlighting quantitative and high-throughput analyses of exosome contents.^19^ Membrane filters can also be incorporated in microfluidic devices to filter exosomes and other EVs. Vesicles in blood samples were collected by sieving driven either by pressure or electrophoresis.^63^ The latter was applied to separate proteins from vesicles based on their distinctly different surface charges. Trapping of exosomes and other vesicles with similar size in microfluidic channels can be achieved based mainly on the difference in size of the components in a sample. By incorporating a porous ciliated silicon microstructure, Wang *et al.* demonstrated a microchip that selectively traps exosome-like lipid vesicles 40–100 nm, while sieving out proteins and cellular debris.^32^ The trapped vesicles were released and collected by simply dispersing the porous structure into PBS buffer.^119^

As mentioned above, the exosome isolation techniques are based on particular properties of exosomes, each with its advantages and disadvantages. Combining two or more technique could further improve the isolation of exosomes^61^ (Table 1).

## Engineering Exosomes as a Therapeutic Delivery System

Exosomes play significant and diverse roles in intercellular communications, particularly in long-distance intercellular signaling. This mechanism of communication is highly robust and efficient in exchanging information between cells.^20^ As such, intact exosomes derived from certain cells possess desirable therapeutic activity.^64^ For example, tumor-derived exosomes that carry specific antigens have been explored for the promotion of specific immune responses against tumors.^2^,^18^ However, it was later found that tumor-derived exosomes can also suppress the immune response and promote metastasis and drug resistance development, shifting the research focus in using exosomes for cancer vaccination towards activating antigen-presenting cells.^74^,^133^ For example, exosomes derived from dendritic cells (DCs), which include peptide-MHC complexes that can be transferred to recipient cells, have been extensively tested for tumor vaccination.^3^,^127^ Intact exosomes derived from human NK cells have been demonstrated to cause tumor cell lysis.^47^

**Figure 2:**
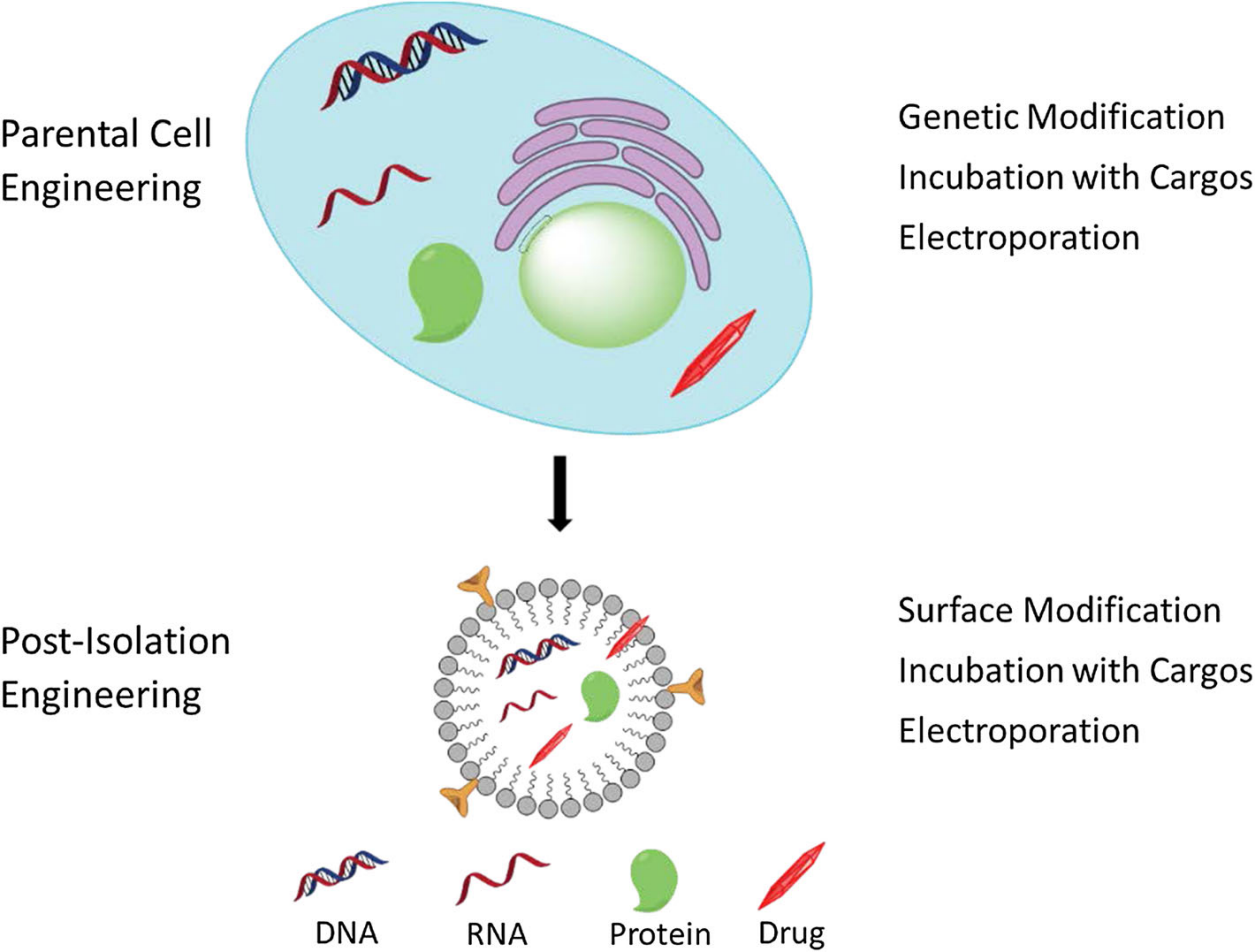
Illustration of exosome engineering through parental cells (upper) and post isolation (lower). Parental cells can be genetically modified to express desirable therapeutic protein or nucleic acids. Drugs can be encapsulated inside parental cells simply by coincubation or electroporation. The exosome surface can be modified with imaging or therapeutic molecules by chemistry or other conjugation methods. Hydrophilic drugs can be encapsulated inside exosomes *via* simple co-incubation or electroporation while hydrophobic drugs are inserted into the hydrophobic core of the lipid bilayer membrane of exosomes.

Inspired by their physiochemical properties and natural cargo-delivering capability, researchers have also explored the potential of exosomes to deliver various exogenous therapeutics.^7^,^73^ Naturally derived from the body, exosomes are able to avoid phagocytosis, fuse with the cell membrane, and bypass the engulfment by lysosomes while causing only limited immune response.^11^,^33^ Exosomes can be engineered to present various targeting/therapeutic molecules on their surface, incorporating hydrophobic compounds in the lipid bilayer membrane and, encapsulating hydrophilic compounds or macromolecules inside their aqueous core. Engineering of exosomes can either be carried out on the parental cells which will secrete exosomes carrying the desirable therapeutics, or directly on the exosomes after they are isolated^42^,^51^ (Fig. 2).

### Engineering of Parental Cells

Due to the availability of various cellular engineering methods, most modifications of exosomes have been performed on parental cells which are then cultured to secrete modified exosomes.^69^,^132^ Approaches to engineer parental cells include: (A) transfecting/infecting parental cells with DNA encoding therapeutically active compounds which are then released *via* exosomes or (B) loading parental cells with a drug, which is then released in exosomes.^7^

#### Transfection and Activation

Protein sequences, along with different types of RNA, are frequently used for cell transfection, ultimately altering the phenotype of the released exosomes.^5^ Genetic engineering strategies may require the use of the calcium phosphate or lipid method (i.e., *via* Lipofectamine) to load cargo or result in the desired genetic expression.^5^ By altering the synthesis of the exosomes, one can control the therapeutic cargo that they will carry.^5^

##### Protein Expression

TRAIL is a therapeutic that has been loaded into cells *via* transfection.^91^,^135^ TRAIL, or TNF-related apoptosis inducing ligand, is a cancer therapeutic that targets death receptors 4 and 5 on cancer cells, ultimately inducing apoptosis.^78^,^121^ In one study, TRAIL-containing exosomes were created by transducing k562 leukemic cells with TRAIL.^91^ The resulting exosomes enhanced apoptosis for melanoma and lymphoma cells. Additionally, MSCs were engineered to create exosomes with TRAIL, which also resulted in the apoptosis of various cancer cell lines, including lung, mesothelioma, breast and renal.^135^

Transfection has been employed to alter exosomes derived from murine immature dendritic cells (DCs). For pre-isolation, immature DCs were transfected with a viral vector to express Lysosome-associated membrane protein 2 (Lamp2b) fused to the αγ integrin-specific iRGD peptide, a membrane protein of exosomal origin, to enhance targeting efficiency to the tumor site.^110^ Post-isolation, exosomes were loaded *via* electroporation with doxorubicin (Dox) and injected intravenously into the murine model, which resulted in reduced breast cancer tumor growth.^110^

##### DNA/RNA

One way that exosome transfection has been accomplished is through miRNA expression vectors, which can result in exosomes carrying miRNA.^5^ In the past, miRNA has been added to exosomes by transfecting miR-143 into THP-1 macrophages.^138^ This modified form of miRNA is then overexpressed in parental cells, resulting in passive loading of the miRNA into exosomes.

For harnessing exosomes for cancer treatments, a study was performed by modifying an invasive triple negative breast cancer cell line (Hs578T) to overexpress miR-134.^84^ The exosomes containing the miRNA were isolated and then used to decrease expression of Hsp90. The miR-134 delivery reduced cell migration and increased the therapeutic efficacy of anti-Hsp90 treatments to the cells.^84^

Similarly, in an effort to analyze the efficacy of exosomes as carriers for anti-tumor microRNAs, Katakowski *et al.*, used electroporation to transfect marrow stromal cells with a miR-146b expression plasmid.^52^ Male Fischer rats were injected intratumorally with the marrow stromal cell-derived exosomes 5 days after glioma injection. Glioma growth was significantly reduced in the rats treated with exosomes containing miR-146b.

To heighten the sensitivity of Hepatocellular carcinoma (HCC) cells to chemotherapeutic agents, it was explored whether exosomes could be delivered to enhance expression of miR-122, a microRNA found to increase the chemosensitivity of HCCs.^138^ Adipose tissue-derived MSCs (AMSCs) were transfected with plasmids containing has-miR-122 using Lipofectamine 2000, or a control plasmid of cel-miR-67, which for human cells contains no mRNA-binding targets. Exosomes were isolated from the supernatant of the AMSCs, and classified as either 122-Exo or 67-Exo depending on their origin and contents.* In vitro*, HepG2 and Huh7 hepatoma cells were exposed to 122-Exo and chemotherapeutic drugs, revealing a decrease in cell viability when compared to controls. *In vivo*, intratumoral injections of 122-Exo increased the sensitivity of HCCs to sorafenib, as observed by reduced tumor size in the mouse model when compared to controls.^67^

Exosomal delivery was also used to deliver microRNA to breast cancer cells expressing epidermal growth factor receptor (EGFR).^141^ Donor cells were transfected with a plasmid to express a fusion of the transmembrane domain of platelet-derived growth factor receptor (PDGF-R) and the GE11 peptide, which binds to EGFR. GE11-positive cells were transfected *via* lipofection with miRNA let-7a, a microRNA reduced in various cancers including breast cancer, whose expression suppresses tumor growth. Exosomes isolated from these cells were intravenously injected into mice with HCC70 breast cancer, successfully delivering the microRNA to the tumor site, as evidenced by reduced tumor growth when compared to control.^85^

Viral packaging can result in exosomes loaded with nucleic acids. This method employs Adeno-associated vectors (AAV) to load exosomes with a viral vector, and has been termed as “vexosomes” (vector-exosomes) in a study by Maguire *et al.*^71^ Human 293T cells were transfected with an AAV plasmid for 48 h, with the resulting vexosomes isolated and collected. The exosomes contained AAV capsids for both strains of the AAV (AAV1 and AAV2), and were subsequently used to deliver DNA to a human glioblastoma cell line U87.^71^

A major benefit of exo-AAV is that they are able to evade neutralizing antibodies, compared to wild type AAV vectors that have the potential to evoke an immune response that can block their delivery.^12^ With difficult-to-target cancer-types, such as glioblastoma, this benefit can be especially useful. Viral exosome delivery was used in the treatment of the GL261 mouse glioblastoma (GBM) model to determine its efficacy as a potential treatment for GBM.^117^ As described previously, exo-AAV were made by transfecting human 293T cells with an AAV plasmid for 48 h.^71^ The role of the vectors was to genetically modify the target GBM cells, causing GFP expression to specifically target Tumor-Associated Macrophages/Microglia (TAMs) and reactive astrocytes. The resulting pathway expressed interferon beta, a cytokine destructive to the brain tumor stromal cells, and was found to increase survival when compared to controls.^117^ The ability of exo-AAV to evade neutralizing antibodies is also beneficial in that it allows for reduced dosing for gene therapy applications.^46^

#### Loading of Exogenous Cargo

Exosomes can be preloaded with a protein or drug of choice when parental cells are loaded with exogenous cargo. The biogenesis process results in the preloading of the exosomes released from the cells, and is especially beneficial for oligonucleotides.^5^ For instance, the parental cells can be incubated along with a specific drug.^69^ The advantage of this strategy is in its simplicity, although there can be issues such as low efficiency of loading and a concern for cytotoxicity of the drug to the cells.^5^

##### Protein

Extracellular vesicles, characterized as exosomes, were conditioned to bear heat shock proteins (HSPs) and isolated from resistant hepatocellular carcinoma cells (HepG2) that had been previously treated with resistant or sensitive anti-cancer cells.^70^ HepG2 cells were incubated at 43 °C, with the HSP-bearing exosomes collected from the media. These exosomes stimulated the cytotoxicity of NK cells and production of granzyme B, and upregulated inhibitory receptors while downregulating activation receptors. Exosomes derived from resistant anticancer drugs were greater in number, with more bearing HSPs, thus increasing the cytotoxic response of NK cells.^70^

Liposomes formed the basis of a study involving exosome delivery of proteins to cancer cells, where they were co-incubated with a murine melanoma cell line (B16BL6).^27^,^28^ The liposomes used for co-incubation were either fluid DOPE-based (1,2-dioleoyl-*sn*-glycero-3-phosphoethanolamine) or solid HSPC-based (hydrogenated soy phosphatidylcholine). Exosomes were collected and isolated from the culture media, their protein contents analyzed and broken up into categories (tetraspanins, heat shock proteins, enzymes and others). The resulting exosomes were then delivered to the B16BL6 melanoma cell line, as well as a murine colorectal cancer cell line (C26), and analyses were performed for studying how exosomal expression of the different proteins impacted their uptake by cancer cells.^27^ The ability to incubate liposomes with cancer cells to produce exosomes with specific proteins could be highly useful for therapeutic delivery.

##### Hydrophilic Drugs

Drug preloading for exosomal delivery of cancer treatments can be accomplished *via* “liposome-based cellular engineering”, which attempts to engineer parental cells *via* membrane fusogenic liposomes (MFLs).^60^ The MFLs have been used to deliver hydrophilic and lipophilic agents into the membrane and cell cytosol. Their resulting extracellular vesicles, which included exosomes and microvesicles, were functionalized to have specific contents without compromising their internal properties. These were able to successfully reduce cancer cell viability compared to control.^60^

##### Hydrophobic Drugs

Macrovesicles (MVs) and exosomes secreted from these MSCs have been frequently studied for therapeutic benefits in regenerative medicine due to their paracrine secretions, in the form of extracellular vesicles such as MVs and exosomes.^53^ In a study of MSCs for use in drug delivery for cancer therapeutics, MSCs were engineered to deliver MVs, and specifically exosomes, with encapsulated paclitaxel (PTX). In this study, PTX priming of the murine MSC cell line SR4987 occurred with a high dosage (2000 ng/mL) of the drug. Researchers found that the MVs released by the MSCs were largely composed of exosomes, which contained PTX. Their delivery* in vitro* and* in vivo* to the human pancreatic adenocarcinoma cell line (CFPAC-1) inhibited cancer cell growth and proliferation both* in vitro* and* in vivo*.^88^

As in the case of hydrophilic drug delivery, membrane fusogenic liposomes can also be used for the delivery of hydrophobic drugs. This method of parental cell engineering was used for anti-tumor drug loading when EVs containing the chemotherapeutics PTX and tirapazamine were co-incubated in Transwell experiments with B16F10 (melanoma) or MDA-MB-231 (late-stage breast cancer) cells. MFLs were able to reduce cell viability when compared to controls, demonstrating the efficacy of preloaded anti-tumor exosomes *via* liposome delivery to parent cells.^60^

### Post-isolation Engineering

In situations where engineering of exosomes at the cellular level is not feasible, exosomes derived from various origins can be engineered to carry functional molecules after being isolated.^62^,^69^ The liposome-like structure of exosomes provides different modification strategies that have been used for liposome modification. The type of cargos to be encapsulated often dictates their loading methods.^11^ Therapeutic cargos that can be loaded into isolated exosomes include small molecules, nucleic acids and proteins.^64^ Macromolecules for targeting, imaging or therapeutic purposes can be conjugated with exosome surface molecules *via* valence bond or other specific conjugation methods.^98^,^120^ Hydrophobic compounds or hydrophilic compounds with a lipid-like hydrophobic tail can be inserted into the hydrophobic core of the lipid bilayer membrane.^33^ Post-isolation modification of one or more exosome structural components falls well within the scope of nanotechnology which have been demonstrated as promising applications in biomedicine, particularly in cancer therapy.^42^,^104^

#### Surface Modification

A few reports have demonstrated the feasibility of exosome surface modification *via* chemical methods.^98^,^105^,^120^ Post-isolation modification of exosome surface structures allows for exosome imaging and tracking* in vivo*.^14^,^102^

To label exosomes for an imaging modality, Smyth *et al.* conjugated fluorescent molecules to the surface of exosomes derived from mouse 4T1 breast cancer cells using click chemistry, a highly efficient and widely-used bioconjugation method.^98^ In the study, the amine groups of exosome surface proteins were first functionalized with terminal alkyne groups which were then reacted with a model azide dye, azide-fluor 545. The mild conjugation did not change the size distribution of the exosomes or their adherence/internalization property with recipient cells.^98^ Instead of using chemical conjugation, an alternative way that has proven effective is to take advantage of specific and tight avidin–biotin interactions. Lai *et al.* transfected human embryonic kidney 293T exosomes to express a surface luciferase with a fused biotin domain which was then coupled with fluorescent Alex Fluor® 680-Streptavidin.^58^ Compared to Cell Tracker insertion labeling, the conjugation labeling increased spatial and temporal imaging resolution of exosomes and enabled the tracking of exosome delivery to tumor sites* in vivo* and analysis of their blood circulation life.^58^

Genetic engineering has also been reported as a method to modify the surface of exosomes.^132^ In one study, exosomes were engineered to express iRGD-Lamp2b to target human breast cancer cell lines, for the delivery of chemotherapeutic agents.^110^

#### Loading of Exogenous Cargo

Exosome membranes can be loaded with hydrophobic therapeutics to increase drug solubility and stability while hydrophilic therapeutics such as RNA can be encapsulated in exosomes to improve cellular delivery.

##### Hydrophobic Drugs

Similar to their passive encapsulation into parental cells, hydrophobic drugs can be inserted into the membrane simply by incubation with exosomes. One of the earliest studies of this kind is the exosomal delivery of curcumin, an anti-inflammatory agent.^103^ In this study, curcumin was mixed with mouse tumor cell line EL-4-derived exosomes at room temperature before the exosomes were purified *via* sucrose gradient centrifugation. Characterization of curcumin-loaded exosomes revealed higher solubility, stability, and bioavailability than free curcumin. In another study, the same group loaded JSI-124, a potent inhibitor of JAK/STAT3 signaling pathway with anti-tumor activity, into EL-4 exosomes, suggesting the potential of exosomes as a general delivery vehicle for hydrophobic compounds.^103^ This incubation method was also used to encapsulate PTX, a hydrophobic chemotherapy drug, and rhodamine 123, a hydrophobic fluorescent compound into exosomes derived from brain tumor cells and endothelial bEND.3 cells for their intranasal delivery across the blood–brain barrier.^56^,^88^,^131^ To combine multiple therapeutic modes in one single nanoscale construct, gold nanorod (GNR)-conjugated Dox was also incorporated into the exosome membrane by simple incubation.^100^ As revealed by TEM, the multiple GNRs were successfully incorporated into the lipid bilayer of the exosome membrane. However, no separation of exosomes and non-encapsulated GNRs was described in the study.^100^

To develop exosome-encapsulated PTX to overcome multiple drug resistance (MDR) in cancer cells, Batrakova’s group compared three different encapsulation methods, incubation at room temperature, electroporation, and sonication to encapsulate PTX into exosomes derived from mouse macrophage RAW 264.7 cells.^55^ Excess free drug was removed by SEC. As measured by HPLC, reformation of the exosomal membrane upon sonication resulted in the highest loading efficiency.^55^

##### Hydrophilic Drugs

Electroporation is more commonly used to load small hydrophilic compounds or nucleic acids into isolated exosomes.^69^ To demonstrate targeted drug delivery for cancer therapy with low immunogenicity and toxicity, Tian *et al.* dispersed purified exosomes derived from an immature mouse DC line in a Dox solution before electroporation was applied.^110^ The mixture was then incubated at 37 °C for 30 min to allow the plasma membrane of the exosomes to recover. After purification by ultracentrifugation to remove unincorporated drug, the encapsulation efficiency reached 20% and the Dox-encapsulated exosomes were able to target and accumulate in breast tumors in mice and inhibit their growth.^110^

##### RNA

As exosomes naturally deliver nucleic acids to recipient cells, exosomes have been expected to deliver exogenous siRNA in an efficient and targeted manner. Similar to their loading into cells, loading of siRNA into exosomes can be achieved by electroporation. Alvarez-Erviti *et al.* reported the first siRNA delivery by isolated exosomes in 2011.^1^ To target the exosomes to the brain of mice, DCs were transfected to express Lamp2b (an exosomal membrane protein) fused to the neuron-specific RVG peptide3. Electroporation was performed on purified exosomes to load exogenous siRNA. Intravenously injected RVG-targeted exosomes delivered the siRNA specifically to neurons, microglia, oligodendrocytes in the brain, resulting in a specific gene knockdown.^1^ Exosomes were also used to deliver siRNA targeting Parkinson’s disease.^89^ Exosomes derived from mouse DCs were loaded with α-synuclein siRNA to target α-synuclein aggregates in the brain in mice with Parkinson’s disease. Brain-specific uptake was observed following intravenous administration of the α-synuclein siRNA-loaded exosomes. Protein aggregates were found reduced at 1 week after injection. This study further supports the feasibility of using exosomes as nanocarriers for transporting cargo across the blood–brain barrier.^89^ To demonstrate that nucleic acids can be delivered across the cell plasma membrane, the same group of authors tested different methods to load RNA into human exosomes of various origins and identified electroporation as the best method for RNA loading. The siRNA-loaded exosomes effectively delivered siRNA into lymphocytes and monocytes, and silenced mitogen-activated protein kinase 1 selectively.^118^ Momen-Heravi *et al.* demonstrated that B cell-derived exosomes can deliver an exogenous miRNA-155 mimic into hepatocytes or macrophages to inhibit malignant growth. Unlike in parental B cells, baseline levels of miRNA-155 was found to be very low in B cell-derived exosomes. The authors optimized the loading efficiency of miRNA-155 by electroporation at various RNA-to-exosome ratios. Exosomes loaded with miRNA-155 mimic significantly increased miRNA-155 levels in primary mouse hepatocytes and the liver of miRNA-155 knockout mice.^79^

## Anti-metastatic Applications

As mentioned above, previous reviews have summarized exosome engineering to target various diseases, in particular cancer.^7^,^11^,^69^,^73^,^104^,^126^,^127^,^132^,^141^ In this section, we introduce the most recent reports of engineered exosomes for metastasis targeting, and their potential for clinical translation.

Phase I clinical trials have demonstrated the feasibility of large-scale production of DC-derived exosomes and the safety of the exosomes in patients with colorectal cancer, lung cancer, and melanoma.^24^,^29^,^80^ In the lung cancer trial, T cell immune responses were detected in one third of exosome-treated patients and increased NK lytic activity in half of the treatment group. Some patients exhibited long term stability of disease and activation of immune effectors. Mouse studies also revealed that the DC-derived exosomes promoted IL-15Rα- and NKG2D-dependent NK cell proliferation and activation respectively, resulting in anti-metastatic effects mediated by NK1.1 (+) cells. In humans, DC-derived exosomes were found to express functional IL-15α which allows proliferation and NK cell secretion of IFN-γ, a cytokine that is critical for innate and adaptive immunity.^80^

Encouraging results were also obtained in a Phase II trial testing DC-derived exosomes as maintenance immunotherapy after induction chemotherapy in patients with metastatic lung cancer.^9^ The exosomes used in this trial were derived from IFN-γ maturated DCs because such exosomes induce greater T cell stimulation compared to those from immature DCs. The exosome treatment increased NKp30-dependent NK cell function in treated patients, and 32% of patients experienced stabilization for at least 4 months.^9^ This Phase II trial confirmed that DC-derived exosomes could boost the NK cell arm of antitumor immunity in patients with metastatic lung cancer.

A promising option in cancer immunotherapy is active vaccination with autologous DCs loaded with tumor-associated peptides. However, the immune response of pancreatic cancer (PaCa) by this strategy is often found suppressed. To overcome this issue, Xiao *et al.* combined vaccination with tumor exosome-loaded DCs (DC-TEX) with drugs affecting myeloid-derived suppressor cells (MDSC). In the study, autologous DCs were loaded with PaCa cell-derived exosomes to vaccinate for PaCa in xenograft mice together with drugs such as Gemcitabine (GEM), all-transretinoic acid (ATRA) and sunitinib.^128^ A reduction of MDSC including tumor-infiltrating MDSC and a decrease in migrating and metastasizing tumor cells was observed in the groups treated with Sun, ATRA and, most efficiently, GEM. Vaccination by DC-TEX with any of the three drugs increased the number of activated T cells in the tumor and subsequently the survival time in mice compared with those vaccinated only by DC-TEX. A reduction in metastatic spread was observed with the combination of (DC-TEX) with sunitinib compared to the group treated with sunitinib alone.^128^

The mutant form of the GTPase KRAS is a key driver of PaCa, which controls macropinocytosis in PaCa cells and increases exosome uptake. This led Kamerkar *et al.* to develop exosomes derived from normal fibroblast-like mesenchymal cells to carry siRNA specific to oncogenic KRAS. Compared to control liposomes, the engineered exosomes exhibited an enhanced retention in circulation and a subsequent enhanced efficacy that is dependent on CD47. It was confirmed that the enhanced retention of exosomes is due to CD47-mediated protection of exosomes from phagocytosis by monocytes and macrophages. The engineered exosomes suppressed cancer in multiple mouse models of PaCa and significantly increased their overall survival. To take this discovery into human translation, the same lab reported bioreactor-based procedures employing good manufacturing practice (GMP) standards to generate large scale production of siRNA-loaded clinical-grade exosomes. The clinical-grade GMP exosomes were tested in multiple* in vitro* and* in vivo* studies to confirm suppression of oncogenic *Kras* and an increase in the survival of several mouse models with PaCa.^50^ The authors also demonstrated that the treatment efficacy could be further improved when combined with the chemotherapy drug gemcitabine.^76^

To overcome MDR in cancer cells, PTX-loaded exosomes were used to treat a drug resistant cancer cell line, MDCK_MDR1_ and its sensitive counterpart MDCK_WT._^55^ In both cell lines, the loading of PTX into exosomes significantly increased drug cytotoxicity compared to PTX alone, with a greater increase in resistant cell lines than sensitive ones. The same researchers also developed macrophage-derived exosomes for targeted PTX delivery to pulmonary metastases.^55^ The drug-loaded exosomes were modified with an aminoethylanisamide-polyethylene glycol vector moiety to target the sigma receptor, which is overexpressed by lung cancer cells. The exosome formulations showed a dramatic ability to accumulate in cancer cells following systemic administration in a C57BL/6 mouse lung cancer model, with improved therapeutic outcomes.

## Conclusions

Exosomes are specialized intercellular messengers that alter the functional state of their target cells by delivering cargo such as proteins and nucleic acids from their parental cells. The role of exosomes in cancer including metastasis has been intensively investigated. Understanding of their properties and activities have provided a solid foundation to engineer exosomes for the targeting of metastasis, which could significantly increase survival among cancer patients. After a decade of research, many engineered exosome engineering methods including those for isolation and cargo incorporation have proven to be successful for modifying exosomes with desirable diagnostic and therapeutic functionalities. Application of engineered exosomes to target metastasis have yielded encouraging results that support further development toward clinical practice.
